# Time-Delayed Subsidies: Interspecies Population Effects in Salmon

**DOI:** 10.1371/journal.pone.0098951

**Published:** 2014-06-09

**Authors:** Michelle C. Nelson, John D. Reynolds

**Affiliations:** 1 Earth to Ocean Research Group, Department of Biological Sciences, Simon Fraser University, Burnaby, BC, Canada; 2 Raincoast Conservation Foundation, Sidney, BC, Canada; North Carolina State University, United States of America

## Abstract

Cross-boundary nutrient inputs can enhance and sustain populations of organisms in nutrient-poor recipient ecosystems. For example, Pacific salmon (*Oncorhynchus* spp.) can deliver large amounts of marine-derived nutrients to freshwater ecosystems through their eggs, excretion, or carcasses. This has led to the question of whether nutrients from one generation of salmon can benefit juvenile salmon from subsequent generations. In a study of 12 streams on the central coast of British Columbia, we found that the abundance of juvenile coho salmon was most closely correlated with the abundance of adult pink salmon from previous years. There was a secondary role for adult chum salmon and watershed size, followed by other physical characteristics of streams. Most of the coho sampled emerged in the spring, and had little to no direct contact with spawning salmon nutrients at the time of sampling in the summer and fall. A combination of techniques suggest that subsidies from spawning salmon can have a strong, positive, time-delayed influence on the productivity of salmon-bearing streams through indirect effects from previous spawning events. This is the first study on the impacts of nutrients from naturally-occurring spawning salmon on juvenile population abundance of other salmon species.

## Introduction

Movement of nutrients across ecosystem boundaries can contribute to the productivity of recipient ecosystems [1]–[2]. This can have a wide range of effects, including individual condition and growth [3], population abundance and distribution [4], and community dynamics [5]–[6]. Subsidies are particularly important to nutrient-limited systems, such as desert islands [7], temperate lakes [8], and freshwater streams [9].

The annual influx of spawning salmon (*Oncorhynchus* spp.) along the temperate coasts of the northern Pacific Ocean constitutes a substantial contribution of marine-derived nutrients to nutrient-poor freshwater streams and lakes [10]–[11]. At the same time, the engineering effects of salmon spawning activities and the marine outmigration of salmon offspring result in some nutrient export [12]–[13]. Reductions in salmon populations in the North Pacific region, which are as high as 95% in some areas [14], have created concern that reduced nutrient availability or streambed engineering by spawning fish may alter the species and communities in freshwater and adjacent terrestrial ecosystems. In fact, the decline of Pacific salmon represents one of the key current environmental issues in North America [15]. Yet without quantifying relationships between salmonids and their ecosystems, it is difficult to inform ecosystem-based management or make holistic management decisions [16].

Since some species of Pacific salmon spend a year or more as juveniles in the same streams that receive nutrients from adult carcasses, it has been suggested that there could be positive feedback across generations of salmon [17]–[18]. For example, coho (*O. kisutch*) spawn far upstream, but juveniles move downstream into areas where high densities of other species of salmon are spawning, such as pink (*O. gorbuscha*) and chum (*O. keta*). Analysis of 8 years of data indicated a positive relationship between the abundance of spawning pink salmon and subsequent spawning adult coho abundance two years later [17]. This idea has taken such a strong hold that it is now common practice for fisheries managers to consider adding salmon carcasses from hatcheries into streams in order to enhance productivity, including growth or survival of juvenile salmon [19]. However, the effects of such a practice have not been rigorously tested. We do know that stream-rearing juvenile salmonids directly consume spawning adult tissue and eggs [18], [20], and they preferentially switch to these resources when they are available [21]. They may also benefit indirectly from spawning salmon nutrients which increase primary productivity [22]–[23] and aquatic and terrestrial invertebrates [22], [24]–[25]. However, bioturbation by large-bodied spawning salmon can also have negative effects on stream invertebrate biomass [26]. Therefore, there remains little evidence of population-level linkages among populations of salmonids.

Nutrients from marine-derived sources, measured by stable nitrogen isotopes, were found in stream salmonids from fall spawning events into the following growing season [27], and marine-derived nutrient signatures were best explained by spawning events in the previous year [28]. While dissolved nutrients are present in the water when salmon are spawning, they do not persist through the non-spawning season [29]. However, stable isotope tracing has shown spawning salmon nutrients are readily taken up by primary producers, aquatic invertebrates [30] and terrestrial invertebrates [25], and these may provide indirect pathways to juvenile coho salmon. Studies have shown increased spawning salmon resource availability is linked to improved condition and growth in juvenile salmonids ([21] Scheuerell et al. 2007), and coho in particular [18], [31]–[32]. However, the effect of spawning salmon on juvenile salmonid abundance is not yet clear, with some studies showing positive effects [18] and others no strong effects [19], [33]. Notably, most previous research has been limited to experimental carcass addition (cf. [31]), which may have different impacts on streams than do live spawning salmon [34].

Abundance of coho juveniles also depends on habitat characteristics, including cover and predator refugia in the form of pools [16], large wood and undercut banks [35]. Coho may also be affected by habitat related to food availability, such as riffle area, fine substrate, gradient [36], and overhead canopy density [33]. Juvenile coho can be limited by physiological tolerances related to temperature [37] and pH [38]. Additionally, stream size is an important predictor of juvenile coho production [39].

In this study we investigate whether juvenile coho salmon benefit from adult pink and chum salmon. Coho spend at least their first year of life rearing in freshwater streams, whereas pink and chum salmon migrate to the ocean within weeks of emerging from the stream substrate [40]. Therefore, juvenile pink and chum have little potential to benefit from salmon nutrients in the stream, whereas their nutrients or engineering effects could affect juvenile coho. Most of the coho that we studied were young of year, and would therefore not have had any direct exposure to spawning salmon in fall at the time of sampling because they emerged only the previous spring. While some egg or tissue consumption may have occurred during the fall sampling period, the juvenile coho would have had at most a few weeks of exposure, thus this is apt to have had minimal effects on population abundance. Coho adults spawn much further upstream in our study streams than pink and chum salmon, and at less than 5% of pink and chum density, so there are likely little to no carcass implications from adult coho.

We conducted a multi-stream comparison to examine the relationship between spawning pink and chum abundance and juvenile coho abundance, and considered a suite of habitat variables that have been shown to be associated with juvenile coho. We also tested whether these habitat variables could have independent effects on the three salmon species. Because the vast majority of coho we sampled were young-of-the-year, any effects would be due to spawning events from previous years. We predicted that chum salmon would have greater effects than pink salmon due to their larger body size and egg deposition [40]. By using naturally-occurring salmon in a wide range of streams, this study encompasses the combination of carcasses, eggs and excreta, as well as engineering effects on the abundance of juvenile salmonids.

## Materials and Methods

### Ethics Statement

All counts of spawning chum and pink, and capture and collection of juvenile coho salmon were approved and conducted in compliance with the guidelines and policies of the Canadian Council on Animal Care (approval number 1021B-07).

### Study sites and design

We surveyed 12 streams on the central coast of British Columbia in the Great Bear Rainforest, in Heiltsuk First Nation traditional territory (Table 1). Pink and chum are the dominant spawning salmon, and juvenile coho were present in all streams. All sites were accessible only by boat. Land use has been very limited in the area, with some selective logging prior to the 1950s [6].

**Table 1:** Stream characteristics, spawning salmon population data (2006–11) and mean juvenile coho abundance (summer and fall, 2008) for streams (n = 12) in this study. Coho salmon abundance and density were log transformed for the analyses.

Stream	Length (m)	Bank full width (m)	Mean pink abundance	Mean chum abundance	Mean coho abundance	Mean coho density (fish/m^2^)
Ada Cove	6,480	11.1	318	1,160	756	0.193
Beales Left	3,360	10.9	1,030	351	1,111	0.367
Bullock Main	2,420	10.9	1,515	2,030	752	0.178
Fanny Left	4,270	12.8	5,008	2,646	48,936	2.97
Hooknose	2,970	16.9	2,970	1,537	13,530	0.632
Jane Cove	1,380	4.6	0	12	214	0.122
Kill Creek	980	3.5	289	797	731	0.505
Kunsoot Main	3,670	13.1	5,800	376	9,272	0.740
Mosquito Left	3,250	4.0	203	92	10	0.006
Port John	2,540	3.3	2	3	164	0.241
Sagar	5,200	15.5	634	779	9,409	0.988
Troup North	440	4.4	1	2	505	0.422

In order to account for the effect of spawning coho adults on the abundance of juvenile coho, it may be helpful to have data for adult coho in streams. However, there were very little historical data available on spawning coho numbers at our streams, nor was it possible to assess this in the field due to the inherent difficulties in estimating spawning coho abundance [41]. However, a consistent relationship between spawning coho and coho smolt abundance has been difficult to find because smolt production is regulated by the availability of rearing habitat in the stream, rather than adult spawning coho abundance (e.g. [39]), unless spawning densities are very low. Furthermore, where data were available within our study area (five streams with spawning coho counts available since 2000), the densities of spawning coho (50–204 females/km) exceed the number of spawning adults that are thought to saturate the habitat with juveniles, which ranges from 4–44 females/km with an average of 19 [42]. Expected juvenile production, calculated as 85 juveniles per spawning female [42] for the five streams (mean  = 11,800) was far in excess of the observed number of juveniles (mean  = 3,592), which further indicates juveniles are limited by something other than spawning coho abundance.

Study streams ranged in bankfull width from 1.2 to 22.8 m, and they all flow directly into the sea. The watersheds range from high gradient exterior coastal sites to lower gradient habitats in coastal fjords. Stream riparian areas are forested within the Coastal Western Hemlock biogeoclimatic zone [43], with a dominant canopy of western hemlock (*Tsuga heterophylla*), western red cedar (*Thuja plicata*), and Sitka spruce (*Picea sitchensis*). Riparian trees and shrubs are dominated by red alder (*Alnus rubra*), salmonberry (*Rubus spectabilis*), salal (*Gaultheria shallon*), false azalea (*Menziesia ferruginea*), and blueberry (*Vaccinium* spp.). Total annual precipitation in the region is amongst the highest in North America, at 3000–4000 mm/yr.

Study streams were sampled for juvenile coho when the pink and chum salmon were spawning in September-October, 2008, as well as prior to spawning in May-June, 2008. Data were available for numbers of adult pink and chum returning to spawn from 2006–2011 across the entire spawning length of each stream. The length of area sampled for environmental variables was scaled to average stream width (30× stream width), and divided into 12 transects. A random subsample of this area was sampled for juvenile coho (8× stream width), as per below.

### Environmental variables

We measured a large set of variables that have been shown or hypothesized to affect abundance of juvenile coho salmon (Table 2). These were: stream catchment area, stream width at bankfull, stream length, maximum stream depth, stream wetted width, large wood, pools, pool:riffle ratio, undercut banks, gradient, canopy cover, percent fines, maximum weekly temperature, pH, and dissolved nutrients (nitrate, ammonia and soluble reactive phosphorous). These variables were combined for model testing (see Data Analysis, below).

**Table 2:** Predictions of the potential influence of habitat features on juvenile coho abundance.

Variable	Mechanism	Direction	References
Stream length	Available habitat increases as stream length increases	Positive	[39]
Stream width	Smaller streams have more structural complexity	Negative	[69]
Large wood	Structures provide cover/predator refuge	Positive	[35]
Undercut banks	Provide cover/predator refuge	Positive	[35]
Pools	Provide cover/predator refuge	Positive	[16]
Pool:riffle ratio	Optimum combination of cover (pools) to invertebrate production (riffles)	Negative outside optimal range	[68]
Fine sediment	Reduces proportion of drift invertebrates, and reduces cover availability by filling spaces between large substrates and structures	Negative	[36]
Gradient	High gradient reduces riffles for intertebrate production, and increases effects of extreme flow events	Negative outside optimal range	[36]
Canopy cover	Provides habitat for terrestrial invertebrates composing drift, but reduces light penetration for primary productivity-feeding aquatic invertebrates	Positive or negative	[2], [33]
pH	Physiological tolerance	Positive (slightly acidic streams)	[38]
Temperature	Physiological tolerance	Negative (for maximum temperatures	[37]

Stream width was measured in two ways. First, we measured the width at water level at the time of sampling, or wetted width. Second, we measured the width at the maximum width without flooding, or bank full width. Both stream width measurements were averaged across 12 transects. Depth was measured at each transect and the highest value used to represent maximum depth. Stream length and catchment area were calculated using iMapBC [44].

Stream temperature was characterized as the maximum weekly average temperature (MWAT) averaged over the two years during which data were collected. Temperatures were measured using two waterproof ibutton data loggers (DS1922L) at two standard transects per stream near the top and bottom of the study reach, which were fastened below the lowest water level to iron rods, and which recorded temperatures every two hours. Water pH was measured at three standard transects per stream throughout the study reach, and ranged between 4.8 and 6.9.

Stream habitat types (pool, riffle, run, glide, rapid) were identified according to Bain and Stevenson [45]. The length and width of each habitat unit was measured, giving a measure of pool:riffle ratio for the stream. Pool depth was also measured at the deepest point, giving an estimate of pool volume for the stream. All pieces of wood that would be in the water at bankfull and which were >10 cm in diameter and >1.5 m long were measured for length and diameter to calculate large wood volume for the stream [35]. Undercut bank percentage for the stream was calculated as the mean length of stream bank undercut on either side, divided by the stream length. Gradient was measured using a clinometer, and vegetative cover using a spherical densitometer at 12 transects per stream. Substrate was measured at 12 transects per stream on the intermediate axis on 10 stones along each transect [46], and categorized into fines (0–1.2 cm), gravel (1.3–10.2 cm), small cobble (10.3–14.9 cm), large cobble (15.0–24.9 cm), boulder (>25.0 cm) or bedrock.

Three water samples were collected at three standard transects at each stream throughout the study reach prior to and during spawning for dissolved nutrients. Dissolved phosphorous (soluble reactive phosphorous) and dissolved inorganic nitrogen (ammonium NH_3_^+^ and nitrate NO_3_^−^) were analyzed by personnel at the Fisheries and Oceans Canada Cultus Lake Research Facility following the American Public Health Association methods [47].

### Spawning pink and chum abundance

Visual surveys by observers walking up streams were available from Fisheries and Oceans Canada for spawning pink and chum abundance at half of the streams in this study between 2006 and 2011 while this study was being undertaken. These data were supplemented using the same survey protocol in partnership with the Heiltsuk First Nation's Integrated Resource Management Department. Fish in all streams were counted for at least two years and up to six years (2006–2011) by either Fisheries and Oceans Canada, Heiltsuk First Nation's Integrated Resource Management Department or Simon Fraser University staff, with an average taken (sum of spawning salmon counts/number of times counted) in order to generally characterize each stream. Because we hypothesized the potential indirect effects from spawning pink and chum salmon to juvenile coho salmon may be on a time scale of longer than one year, we have elected to use mean 2006–2011 adult pink and chum abundance rather than the spawning year prior to sampling. However, results were similar using only spawning pink and chum abundance for 2007.

At least three spawning salmon counts were undertaken at each stream in each spawning season. The total abundance was estimated using the area-under-the-curve method where a curve is created showing abundance over time and the area under that curve used to estimate total abundance [48]. When we could not access the stream three times within a spawning season, the single or the average of two counts were used. There was no substantive difference between methods at a subset of cases using both methods [6].

### Juvenile coho abundance

In May-June and September-October, 2008, juvenile coho were collected by triple-pass depletion of a stop-netted section of each stream. Due to the remoteness of our sites and the complexity of streams, we elected to use a two-meter wide pole seine to collect juvenile coho (e.g. [49]). This involved two people walking upstream, each holding a pole with the seine net stretched vertically perpendicular to the flow of water, and a heavy chain on the bottom of the net reaching the stream substrate. The seine is quickly moved across the substrate and through the water, lifted periodically to a horizontal position, and the coho contained immediately removed with a small dip net. Sampled areas were left undisturbed for a minimum of one hour between passes, with the same methods used for each pass. Sections were chosen randomly within the area sampled for environmental variables with seine section length standardized as 8× bankfull width. In order to ensure stable and representative coho density throughout the entire section, the sampled area included representation from all habitat types (pools, riffles, glides, and runs) with an average area sampled for coho density of 231.9 m^2^. Resulting coho density (juvenile coho/m^2^) was used to calculate abundance (juvenile coho/stream) in the spawning reach for each stream.

Maximum likelihood modeling was used with the three pass depletion data to estimate total abundance [50]. A comparison between a standard multinomial method [51], maximum likelihood [50], and a hierarchical approach [52] for estimating abundance from depletion found no significant difference in abundance estimates between methods (ANOVA, n = 12, p>0.05). As streams were sampled consecutively over a period of six weeks, we tested for an effect of sampling date within season on abundance. No effect was found, therefore sampling date was not included in further analyses within each season.

Age analysis of scales from a small subset of individuals (n = 5 at each stream) revealed the vast majority (87.8% in summer and 81.0% in fall) were young of year (hatched in spring of the same year of sampling) and the remainder hatched the previous spring. We were unable to separate the remaining fish by age class, nor were we able to model abundance for age classes separately, thus our abundance values include both age classes.

### Data analysis

Given the large number of potentially inter-related environmental characteristics assessed (Table 2), we used principal components analysis (PCA) to reduce 17 habitat variables into orthogonal axes. All axes explaining more than 5% of the variance were extracted for further analysis [53]. These axes explained 64.8% of the variation in habitat characteristics among streams in three principal components; watershed size (PC1), habitat structure (PC2), and dissolved nutrients (PC3) (Table S1). The component representing watershed size (PC1) includes catchment area, stream length, bank full width and wetted width, as well as dissolved phosphorous. The component mainly representing habitat structure (PC2) includes percent undercut bank, large wood volume, and gradient, as well as pH. The component representing dissolved nutrients (PC3) includes maximum temperature, dissolved nitrate and dissolved phosphorous (Table S1).

Next, we assessed the relative importance of pink salmon abundance, chum salmon abundance, and the habitat principal components as explanatory variables of juvenile coho salmon abundance in summer and fall. Linear models were constructed to represent our *a priori* hypotheses. Although it is possible habitat characteristics, such as those affecting nutrient retention or availability, may mediate the relationships between spawning pink and chum and juvenile coho abundance [54], we did not include interaction terms in order to avoid over-parameterization [55]. However, preliminary correlation analyses between habitat variables and spawning pink and chum abundance did not reveal strong interactions (r-squared <0.25). A null model was included in each candidate set. To account for the lack of independence from data from 2007 and 2008, we included year as a fixed effect in our models. Coho abundance was log_10_ transformed to reduce over-leveraging of outlying data points.

Akaike's information criterion adjusted for small sample sizes (AICc) was used to evaluate the relative importance of the candidate sets of linear models for juvenile coho abundance as the response variable. AIC evaluates the predictive power of models with different combinations of variables based on the principle of parsimony, which balances optimal fit with the number of variables used in the model [56]. We used all model combinations with a maximum of three variables per model to avoid over-fitting [55]. Candidate models were computed using the maximum likelihood estimation method [57]. We inspected model diagnostics for heteroscedasticity, over-leveraging of data points, and normality and independence of residuals. Model averaging was then used to quantify and rank the importance of individual explanatory variables for each response variable using summed model weights [58]. We incorporated all of the candidate models (including those with ΔAICc>2) into the model averaging for each response variable. ΔAICc values, which represent the difference between model *i* and the top ranked model, are reported for all models with ΔAICc<3 [55], [59].

We wanted to determine whether stream size could drive patterns of juvenile salmon abundance. Therefore, the principal component representing these variables was included in AICc model testing, with coho abundance as the response variable. An alternative would have been to calculate fish densities instead of abundance, i.e. juvenile coho, and spawning pink and chum per unit stream size (Figure S1). We found similar results, and we have chosen to present the abundance results with stream size as a separate parameter in order to see the independent effects of stream size rather than combine it with spawning salmon. We also used partial correlation analysis to determine the unique contribution of pink and chum abundance to coho abundance after the influence of stream size and other habitat characteristics (principal components] had been removed.

All statistical analyses were performed using R [60], including the MuMIn package [61].

## Results

**Figure 1:**
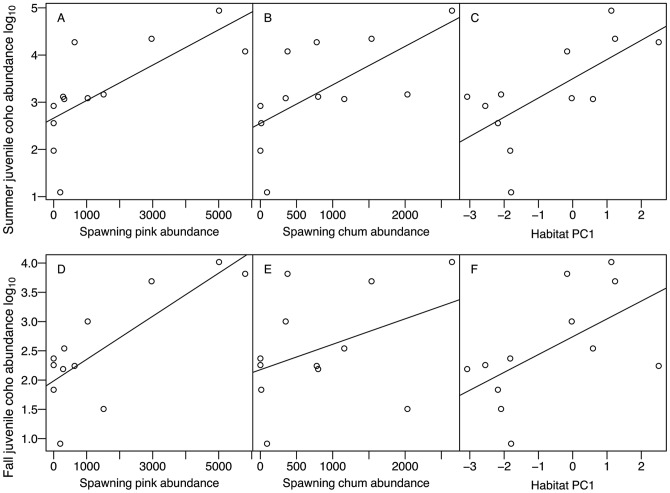
Relationships between the abundance of spawning pink and chum salmon and habitat principal components, and abundance of juvenile coho salmon in summer prior to spawning (a–c) and during spawning in fall (d–f). Large values of PC1 correspond to variables related to large watersheds. Correlation coefficients (r) are in Table 4.

**Figure 2:**
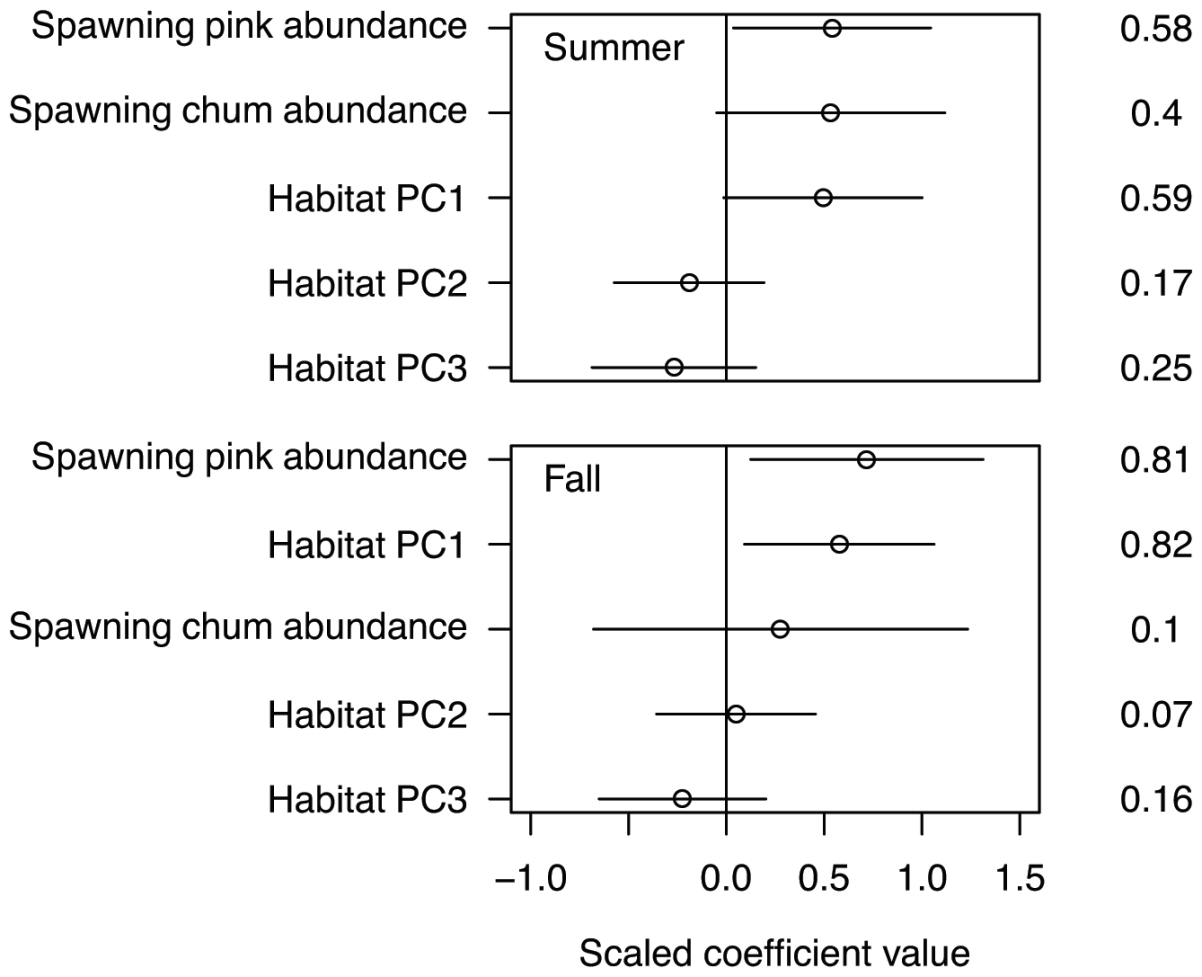
Scaled model parameter estimates (circles) with 95% confidence intervals (lines) from averaged predictive linear models describing juvenile coho salmon abundance in summer (top) and fall (bottom). The variables are ordered from the highest positive scaled coefficient value to lowest negative value. The relative importance of variables to the averaged model (indicated on the right) is scaled from 0 to 1.

High summer juvenile coho abundance was associated with high pink and chum abundance and large watershed size (PC1, Figure 1). These three variables were the only important correlates of summer coho salmon abundance, (ΔAICc<2, relative importance 0.58, 0.4 and 0.59, respectively; Figure 2). After taking the effect of habitat components, including watershed size (PC1), into account, the resulting positive relationship between pink and chum abundance and juvenile coho abundance was still clear (partial r-squared  = 0.35 and 0.55 for pink and chum, respectively). Note that the remaining correlation between chum and coho was stronger than pink and coho when the effect of habitat was controlled statistically, which was consistent with our prediction.

For fall coho abundance, spawning pink salmon abundance and watershed size (PC1) explained differences in juvenile coho abundance better than chum abundance, habitat structure (PC2), or dissolved nutrients (PC3) (Table 3). Every 1,000 pink salmon adults were associated with 1,500 more juvenile coho salmon (Figure 1). The model containing spawning pink abundance and watershed size was the only model with ΔAICc<2 (relative importance  = 0.81 and 0.82 for pink abundance and watershed size, respectively; Figure 2). The relationship between pink abundance and fall coho abundance remains after taking the effect of habitat components into account (partial r-squared  = 0.59), while no relationship remains between chum abundance and coho abundance in fall (partial r-squared  = 0.04).

**Table 3:** Summary of linear regression models with the greatest support (ΔAICc<3.0) for juvenile coho salmon abundance in summer and fall.

	Model parameters	K	R^2^	ΔAICc	w_i_
Summer juvenile coho abundance	Pink + PC1	4	0.73	0.00	0.23
	Pink	3	0.61	1.20	0.13
	Chum + PC1	4	0.68	2.34	0.07
	Chum + PC3	4	0.68	2.38	0.07
	PC1	3	0.57	2.50	0.07
	Chum	3	0.56	2.74	0.06
	Chum + PC1 + PC3	5	0.77	2.91	0.05
Fall juvenile coho abundance	Pink + PC1	4	0.76	0.00	0.50

AICc =  Akaike's information criterion with a correction for small sample size, K =  number of model parameters, R^2^ =  model correlation coefficient, ΔAICc =  change in AICc score from top model, w_i_ =  AICc model weight. The models are ordered by decreasing w_i_.

**Figure 3:**
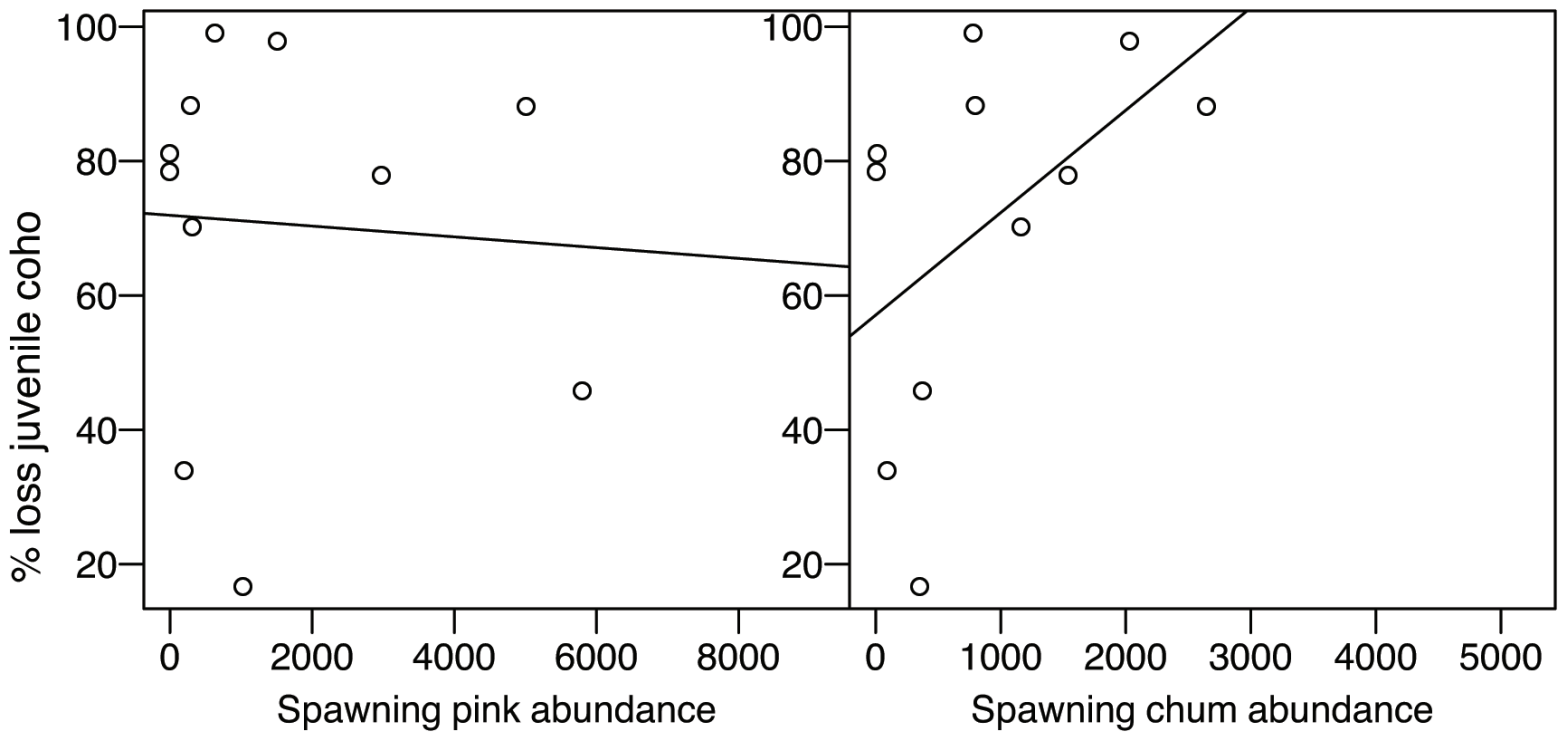
Relationships between the percent loss of juvenile coho salmon between summer and fall and the abundance of spawning pink and chum salmon.

Streams that had the greatest loss of juvenile coho between summer and fall had larger numbers of chum adults (r = 0.49; Figure 3). However, there was no relationship with the abundance of pink salmon (r = −0.06).

The relationships between the broad suite of habitat variables measured (Table 2) and coho abundance were weaker than the relationships between pink and chum abundance and coho abundance (Tables 3 and 4). Pink and chum abundance were also correlated with the percentage of the substrate that was small cobble (r^2^ = 0.52 and 0.48, respectively). Small cobble was not correlated with coho abundance (r^2^<0.1).

**Table 4:** Bivariate correlations, r, between variables used in the analyses. Coho salmon abundance has been log transformed.

	Fall coho abundance	Pink abundance	Chum abundance	Habitat PC1	Habitat PC2	Habitat PC3
Summer coho abundance	0.79	0.78	0.75	0.75	−0.16	−0.21
Fall coho abundance	-	0.76	0.56	0.74	0.02	−0.31
Pink abundance	-	-	0.55	0.61	0.02	−0.16
Chum abundance	-	-	-	0.66	0.08	0.17
Habitat PC1	-	-	-	-	0.00	0.00
Habitat PC2	-	-	-	-	-	0.00

## Discussion

We found that streams containing higher densities of spawning pink salmon had more juvenile coho salmon. Juvenile coho were also more abundant in streams that had more spawning chum salmon, though this was true only in the summer period prior to the arrival of spawning adult chum. Because over 80% of the coho sampled in the pre-spawning portion of this study were recently hatched and had no direct contact with spawning adults of any species, our findings suggest a legacy effect of salmon nutrient subsidies through indirect effects. Other studies have shown marine-derived nutrients to persist in aquatic invertebrates and stream salmonids from fall into summer [27] and a legacy signature of marine-derived nutrients in juvenile coho that is best explained by spawning salmon run size the previous year [28].

Watershed size was as important in explaining juvenile coho abundance as pink and chum abundance, whereas watershed size and spawing salmon abundance were much better at predicting juvenile coho abundance than the broad suite of other habitat characteristics considered. This multi-stream comparison also complements a study of one stream with 8 years of data suggesting that adult coho abundance is positively related to the abundance of adult pink salmon [17].

Several mechanisms may explain the strong and positive indirect effects of spawning pink and chum on juvenile coho abundance. For example, there could be a bottom-up trophic pathway if dissolved nutrients from spawning salmon enhance primary productivity. It is also possible that salmon subsidize invertebrates feeding directly on carcasses, which could be eaten by juvenile coho. Both mechanisms have been shown, with enhanced primary production [22]–[23] and increased invertebrate biomass [22], [24]. Indeed, at the streams in this study, other research has found spawning salmon biomass to be the best predictor of summer biofilm and chlorophyll a, and salmon-derived nitrogen in biofilm to be 2–3× higher in sites below barriers to pink and chum compared to above [30]. Furthermore, at these same streams, spawning salmon biomass was an important predictor of salmon-derived nitrogen and carbon in aquatic invertebrates [62]. Although dissolved nutrients were not strong predictors of coho abundance, they were more strongly related to spawning pink and chum in fall than during summer (Table 4), suggesting these nutrients do not persist in the water for long after spawning events.

Previous studies have tested for impacts of salmon on densities of juvenile salmonids using experimental additions of carcasses. Bilby et al. [18] showed an increase in the density of juvenile coho following the addition of adult coho carcasses to two natural streams. Lang et al. [31] found a general pattern of greater coho density in ponds connected to spawning habitat by hyporheic flow, which is consistent with our findings. Other studies have found no change in juvenile salmonid density with the addition of carcasses to three natural streams [19], [33]. While carcass addition studies can examine the effects of direct consumption of carcass tissue, they do not take into account the full effect of spawning salmon [34], including the influence of nutrient provision in the form of eggs, and these nutrients are readily used by juvenile salmonids [21], [63], nor do they include the effect of dissolved nutrients through excretions [64], or the potential engineering effects of spawning activities [26]. In addition, live fish excrete nutrients that have higher bioavailability than carcasses and may be more effective in stimulating primary productivity, particularly in nutrient-limited systems [64]. Furthermore, older juvenile coho can prey upon newly-hatched pink and chum fry [65].

A potential issue with comparisons of natural variation among streams is that habitat variables could confound the results. For example if all three species of salmon respond in the same way to the same habitat variables, that could lead to spurious correlations. However, by taking a broad range of habitat variables found to be associated with juvenile coho into account explicitly and using an information theoretic model comparison, we have attempted to minimize the chance of this occurring. Specifically, we measured 17 habitat characteristics known to be correlated with abundance of juvenile coho. The relationships between spawning salmon and juvenile coho were stronger than the relationships between any of the three species and habitat characteristics, though the relationship with watershed size was high, which we attempted to isolate using a partial correlation approach. We also note that habitat usage by coho is very different from the others. Adult coho travel much further upstream, and the young spend a year or more in freshwater, favoring pools and large wood structures (Table 2, see also [16], [35]). In contrast, pink and chum salmon spawn lower down in the stream, and their juveniles leave for the ocean immediately after they emerge in the spring.

The relationship between juvenile coho abundance and adult chum salmon was strong in the summer before adults arrived but there was no relationship in the fall, when the fish were spawning. We also found the percent reduction in coho abundance from summer to fall was positively related to chum abundance but not to pink abundance. These effects may be due to more aggressive behavior of chum displacing juvenile coho (personal observation), or stronger bioturbation by chum, which are considerably larger than pink salmon. Although no previous studies have looked at the effect of aggressive behavior of chum on juvenile coho, we do know that juvenile coho may be negatively affected by aggressive behavior of conspecifics. For example, Bradford et al. [42] estimated 60–90% of newly hatched coho become displaced and move downstream into the marine environment in their first spring due to intraspecific aggression and high water flows, resulting in mortality. Furthermore, bioturbation could reduce foraging success of juveniles through reduced invertebrate biomass [26] and thus mediate the positive effect of the nutrient subsidy to primary and invertebrate production [22]–[24], although bioturbation may also increase drifting invertebrates which may increase foraging success of juveniles. Bioturbation can also increase the availability of salmon eggs to other species [21], but only approximately 20% of the coho in our study would have had access to eggs. Further data on primary and invertebrate productivity would be required to fully elucidate the importance of a bioturbation mechanism in our system. Additionally, comparing diets of juvenile coho in summer prior to spawning and fall during spawning may illuminate underlying trophic mechanisms at play.

This study advances our understanding of the strength and persistence of nutrient subsidies in resource-limited systems such as freshwater streams while taking important habitat characteristics into account. There is a great deal of interest in the importance of such cross-ecosystem subsidies in fisheries and ecosystem-based management [6], [66]–[67]. Our results suggest that spawning salmon have indirect but significant influences on stream-rearing juvenile salmonid populations that persist in the environment, creating a legacy effect of marine nutrient subsidy.

## Supporting Information

Figure S1 **Relationships between the densities of spawning pink and chum salmon and habitat principal components, and density of juvenile coho salmon in summer prior to spawning (A–C) and during spawning in fall (D–F).** Large values of PC1 correspond to variables related to large watersheds.
(TIF)

Table S1 **Component loadings of 17 habitat variables for the first three components, which collectively explain 64.8% of the total variance in the data.**
(DOCX)
