# Supplementary figures and images for: Time-Delayed Subsidies: Interspecies Population Effects in Salmon
Source: PLoS One. 2014 Jun 9;9(6):e98951. doi: 10.1371/journal.pone.0098951 (PMC4049634; doi:10.1371/journal.pone.0098951)

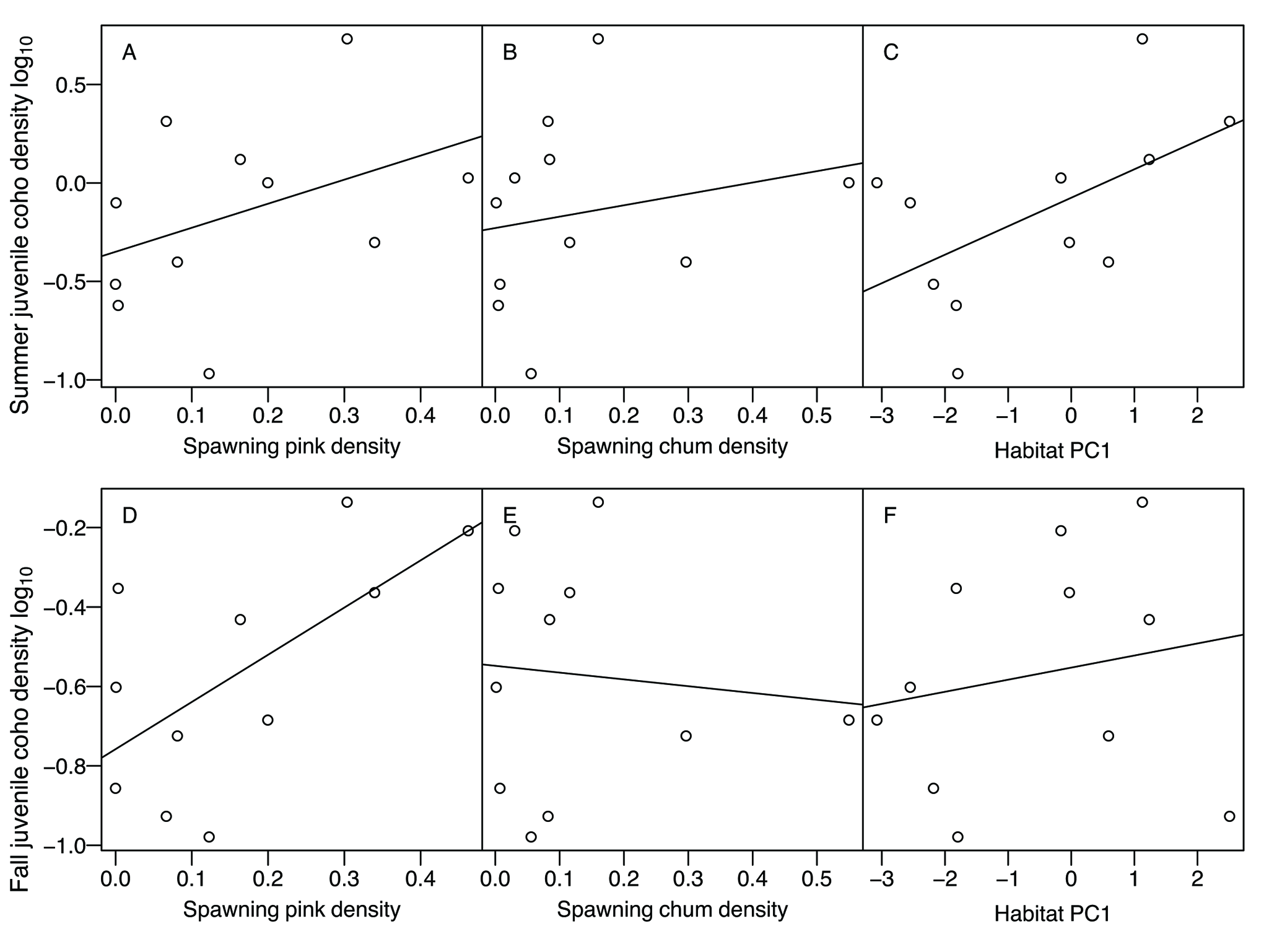

Supplement: Figure S1 — Relationships between the densities of spawning pink and chum salmon and habitat principal components, and density of juvenile coho salmon in summer prior to spawning (A–C) and during spawning in fall (D–F). Large values of PC1 correspond to variables related to large watersheds. (TIF) [file pone.0098951.s001.tif]
