# Supplementary material for: Time-Delayed Subsidies: Interspecies Population Effects in Salmon
Source: PLoS One. 2014 Jun 9;9(6):e98951. doi: 10.1371/journal.pone.0098951 (PMC4049634; doi:10.1371/journal.pone.0098951)
Supplement: Table S1 — Component loadings of 17 habitat variables for the first three components, which collectively explain 64.8% of the total variance in the data. (DOCX) [file pone.0098951.s002.docx]

**Table S1.** Component loadings of 17 habitat variables for the first three components, which collectively explain 64.8% of the total variance in the data.

| Variable | PC1 | PC2 | PC3 |
| --- | --- | --- | --- |
|  | *39.10%* | *14.30%* | *11.40%* |
| Catchment area | **0.388** | -0.017 | 0.041 |
| Stream length | **0.461** | 0.108 | 0.177 |
| Maximum stream depth | 0.256 | 0.047 | 0.019 |
| Stream width at bank full | **0.412** | -0.088 | 0.073 |
| Stream wetted width | **0.432** | -0.079 | 0.067 |
| Percent undercut | -0.079 | **0.341** | -0.37 |
| Pool volume | 0.184 | 0.127 | -0.069 |
| Pool to riffle ratio | 0.018 | -0.005 | -0.067 |
| Large wood volume | 0.029 | **-0.305** | -0.288 |
| Gradient | -0.051 | **-0.543** | 0.009 |
| Percent fines | -0.059 | 0.209 | 0.222 |
| Canopy density | 0.051 | -0.218 | 0.236 |
| Maximum temperature | -0.005 | -0.232 | **-0.564** |
| pH | 0.012 | **-0.523** | 0.241 |
| Dissolved nitrate | -0.094 | -0.169 | **0.472** |
| Dissolved ammonia | -0.18 | -0.07 | -0.154 |
| Dissolved phosphorous | **-0.354** | -0.001 | **0.367** |

*Parameters accounting for most of the variability in each principal component are shown in bold. PC1 is mainly related to watershed size, PC2 to habitat structure and pH, and PC3 to water nutrients and temperature.*
